# Electro-Forming and Electro-Breaking of Nanoscale Ag Filaments for Conductive-Bridging Random-Access Memory Cell using Ag-Doped Polymer-Electrolyte between Pt Electrodes

**DOI:** 10.1038/s41598-017-02330-x

**Published:** 2017-06-08

**Authors:** Myung-Jin Song, Ki-Hyun Kwon, Jea-Gun Park

**Affiliations:** Department of Electronics and Computer Engineering, Hanyang University, Seoul, 04763 Republic of Korea

## Abstract

Ag-doped polymer (polyethylene oxide: PEO) conductive-bridging-random-access-memory (CBRAM) cell using inert Pt electrodes is a potential electro-forming free CBRAM cells in which electro-forming and electro-breaking of nanoscale (16~22-nm in diameter) conical or cylindrical Ag filaments occurs after a set or reset bias is applied. The dependency of the morphologies of the Ag filaments in the PEO polymer electrolyte indicates that the electro-formed Ag filaments bridging the Pt cathode and anode are generated by Ag+ ions drifting in the PEO polymer electrolyte toward the Pt anode and that Ag dendrites grow via a reduction process from the Pt anode, whereas electro-breaking of Ag filaments occurs through the oxidation of Ag atoms in the secondary dendrites and the drift of Ag^+^ ions toward the Pt cathode. The Ag doping concentration in the PEO polymer electrolyte determines the bipolar switching characteristics; i.e., the set voltage slightly decreases, while the reset voltage and memory margin greatly increases with the Ag doping concentration.

## Introduction

CBRAM has been intensively studied as a promising nonvolatile memory cell for storage-class memory and for terabit-integration nonvolatile memory cells^1–3^, because it has a bipolar switching characteristic^4, 5^, is capable of multi-level-cell (MLC) operation^6^, has sufficient write/erase endurance cycles and retention time^7, 8^, and operates quickly^9, 10^. Its nonvolatile memory characteristics originates from ionic conduction through drift and electrochemical redox due to the reactive electrode causing metal ion filaments to form (electro-forming) and break (electro-breaking) in the solid electrolytes between the top and bottom electrode^7, 11–13^. In particular, the reactive electrode plays an important role in determining the nonvolatile memory characteristics. Only oxidative materials can be used as reactive electrodes in CBRAM. Moreover, only a few materials such as Ag^11–13^, Cu^7, 14–17^, and CuTe have been used in the reactive electrodes; i.e., there is a limitation on the usable electrode materials^18, 19^. In addition, it has been reported that the electroforming voltage (*V*_*forming*_) of CBRAM cells is higher than the set voltage (*V*_*set*_), thereby requiring an electro-forming program prior to memory-cell operation, which places a burden on nonvolatile memory operation. To overcome these disadvantages, many researchers have tried to make electro-forming free CBRAM cells^20–23^. Thus, with the goal of overcoming the limitation on usable electrode materials (i.e., Ag, Cu, and CuTe etc.) and achieving an electro-forming free CBRAM cell, we devised a novel CBRAM cell with a sandwiched structure including a top inert electrode (Pt), Ag-doped PEO polymer electrolyte, and a bottom inert electrode (Pt). In particular, we investigated how varying the Ag doping concentration affects the bipolar switching characteristics such as *V*_*forming*_, *V*_*set*_, reset voltage (*V*_*reset*_), high resistance state (HRS), and low resistance state (LRS).

In addition, although morphologies have been reported for conductive metallic filaments in a solid electrolyte of a CBRAM cell, the mechanism behind electro-forming and electro-breaking of the conductive metallic filaments has not been explained through any morphology observation^6, 14, 24, 25^. Thus, we intentionally designed a lateral CBRAM cell using Ag-doped PEO polymer electrolyte between inert Pt electrodes. Then, we investigated the detailed morphologies of the Ag filaments in the PEO polymer electrolyte after a set or reset bias was applied by using top-view secondary-electron-microscopy (SEM) and by conducting a chemical composition analysis using energy-dispersive-x-ray-spectroscopy (EDS) depending on Ag doping concentration. The current conduction mechanism of set (electro-forming of Ag filaments), LRS, reset (breaking of Ag filaments), and HRS could be explained by fitting the results to bi-stable switching behavior. Finally, we examined the correlation of the current conduction mechanism with the morphologies of the Ag filaments in the PEO polymer electrolyte after set and reset biases were applied.

## Results

**Figure 1:**
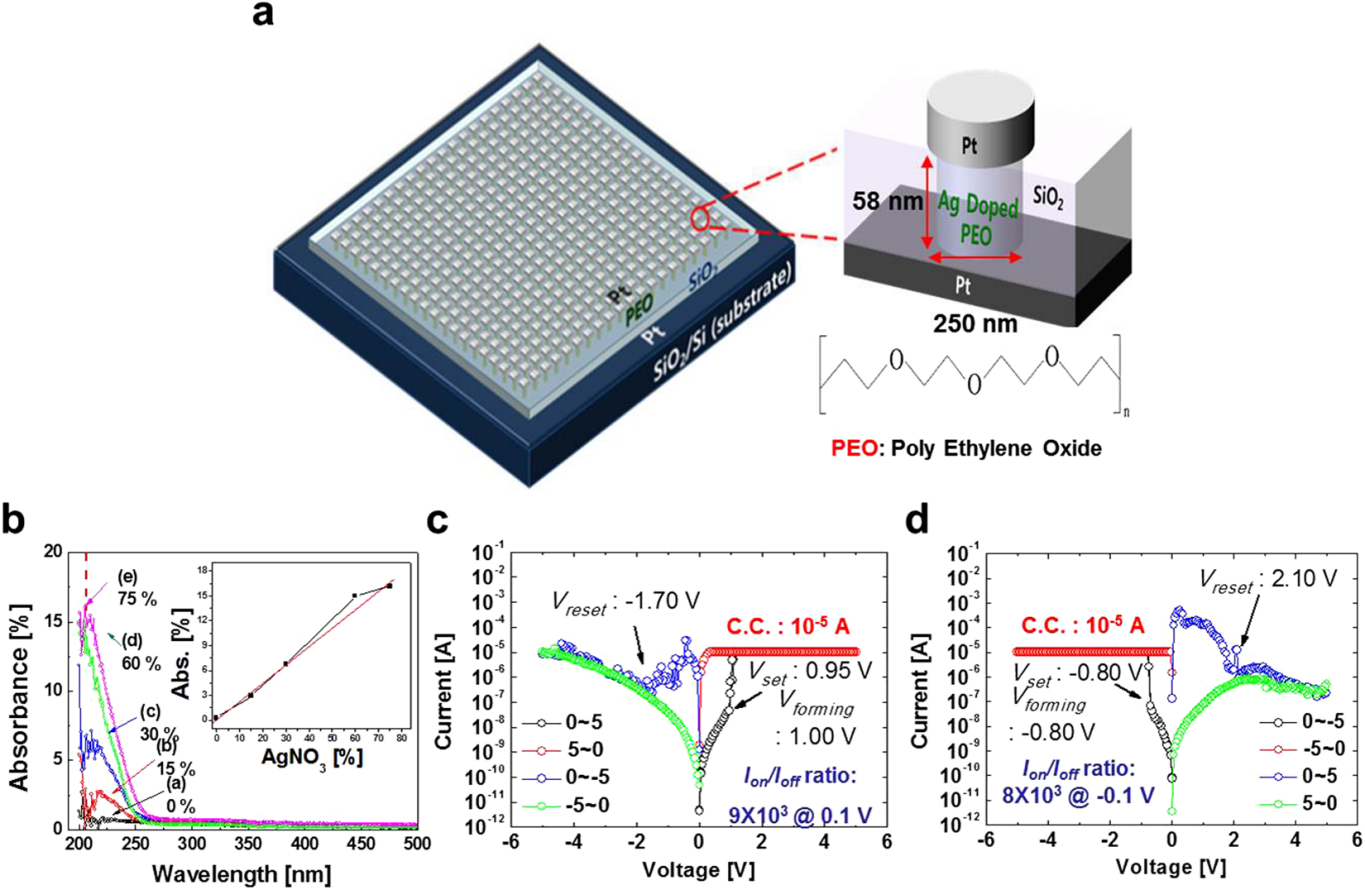
Bipolar switching characteristics of Ag-doped PEO polymer-electrolyte based CBRAM cell. (**a**) Schematic device structure of CBRAM cells, (**b**) dependence of UV absorption on Ag doping concentration in the PEO polymer electrolyte, (**c**) *I–V* curve of CBRAM cell while electro-forming at a positive applied bias, and (**d**) *I*–*V* curve of CBRAM cell while electro-forming at a negative applied bias.

In case of the CBRAM cell with a top reactive and a bottom inert electrode, the set and reset operations can be obtained by applying bias in a specific direction only^26^. Otherwise, due to its symmetric device structure using the same top and bottom electrode material, the set and reset directions of the CBRAM cell with the Ag-doped PEO polymer electrolyte can be determined by the electro-forming direction. For electro-forming at a positive applied bias, the CBRAM has a *V*_*forming*_ of +1.00 V, *V*_*set*_ of +0.95 V, and *V*_*reset*_ of −1.70 V, as shown in Fig. 1c. Remind that V_*forming*_ is determined by being reached at the abrupt current increase (i.e., the compliance current level) at the first time, meaning that conductive filaments were formed in the polymer electrolyte. V_*set*_ is found by arriving at the abrupt current increase the second time, indicating that the current is conducted through Ag filaments in the polymer electrolyte. V_*reset*_ is defined at an applied bias when the current drops from LRS to HRS. In addition, for electro-forming at a negative applied bias, the CBRAM has a *V*_*forming*_ of −0.80 V, *V*_*set*_ of −0.80 V, and *V*_*reset*_ of +2.10 V, as shown in Fig. 1d. These results indicate that the CBRAM cell can perform bi-polar switching in any direction of the electro-forming voltage, unlike other CBRAM cells^27^. In addition, the *I*–*V* curves were not symmetric between electro-forming at a positive and a negative applied bias since the shapes of the conductive filaments in the polymer electrolyte would be different between electro-forming at a positive and a negative applied bias. Note that C.C. determined the *I*–*V* characteristics such as *V*_*set*_, *V*_*reset*_, and *I*_*on*_ */I*_*off*_ ratio for both a negative applied bias and a positive applied bias, as shown in Fig. S1. In addition, the CBRAM cells did not need an electro-forming process, since *V*_*forming*_ was almost equal to *V*_*set*_ unlike previous CBRAM cells in which *V*_*forming*_ is higher than *V*_*set*_. Hence, this cell is electro-forming free^20, 21^. Furthermore, it was confirmed that the DC cycles of greater than 1 × 10^3^ for a positive allied bias was sustained by the Ion/Ioff ratio of 9.41 × 10^3^, as shown in Fig. S2, and the 1 sigma(σ) of *I*_*HRS*_, *I*_*LRS*_, *V*_*forming*_, *V*_*set*_ and *V*_*reset*_ was 4.96 × 10^−9^ A, 2.95 × 10^−6^ A, 0.09 V, 0.11 V and 0.11 V at a positive applied bias and 2.13 × 10^−9^ A, 1.17 × 10^−6^ A, 0.04 V, 0.11 V and 0.17 V at a negative applied bias, respectively, as shown in Fig. S3.

**Figure 2:**
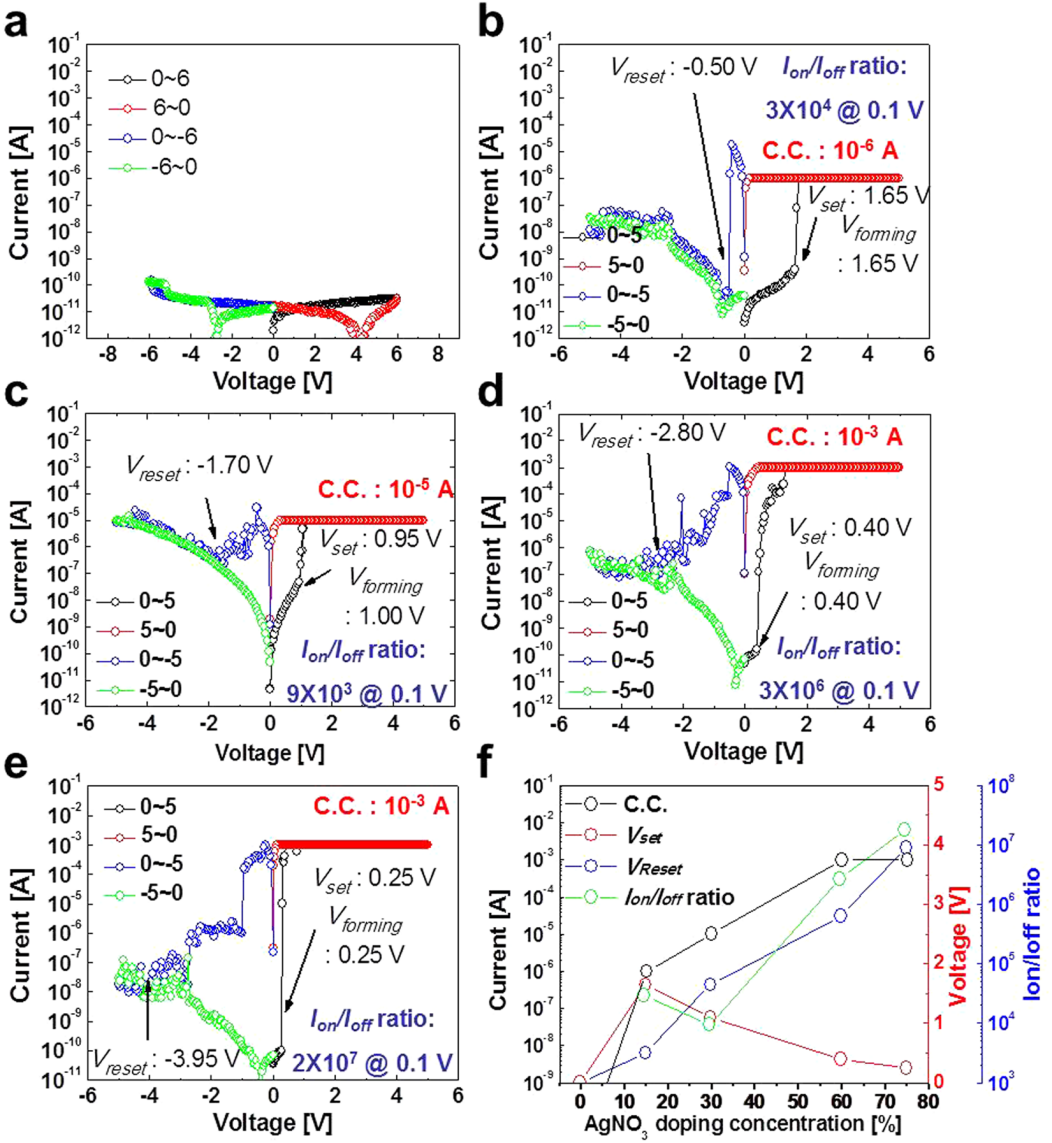
Dependence of bipolar switching characteristics on Ag doping concentration for Ag-doped PEO polymer-electrolyte based CBRAM cells. *I–V curves* for (**a**) 0 wt%, (**b**) 15 wt%, (**c**) 30 wt%, (**d**) 60 wt%, and (**e**) 75 wt%. (**f**) Compliance current level (C.C.), set voltage (*V*_*set*_), reset voltage (*V*_reset_), and memory margin (*I*_*on*_ */I*_*off*_).

Next, we investigated the dependence of the bi-polar switching characteristics on the Ag dopant wt%. Without Ag doping, the CBRAM cell showed no bi-polar switching (Fig. 2a), whereas the Ag-doped cell could perform bi-polar switching. In particular, the cell’s bipolar switching characteristics strongly depended on the Ag dopant wt% (Fig. 2b–e). *V*_*reset*_ increased while *V*_*set*_ decreased with increasing Ag doping wt%, and the compliance current level (C.C.) increased with Ag doping wt% (Fig. 2f). In addition, it was confirmed that the bipolar switching characteristics at a negative applied bias was almost similar to that at a positive applied bias, as shown in Fig. S4. As a result, the memory margin (*I*_*on*_ */I*_*off*_) increased with the Ag dopant wt%. Note that C.C. was determined by being reached at the maximum *I*_*on*_ */I*_*off*_. Furthermore, it was confirmed that the *I*_*on*_ */I*_*off*_ ratio at the DC cycles of ~5 × 10^2^ was dependent of C.C.: i.e., 3.0 × 10^2^ and 2.9 × 10^3^ at 10^−6^ and 10^−5^ A respectively as shown in Fig. S5. This dependence means that Ag dopants in the electrolyte enhance the formation of Ag filaments, and the number and diameter of filaments in the electrolyte increases with the Ag dopant wt%. Here, *V*_*set*_ decreased with increasing Ag dopant wt% while C.C. increased with Ag dopant wt% probably because the larger number and larger diameter of the filaments in the electrolyte more easily produced a conduction path in the cell. In addition, more and larger-diameter Ag filaments would be more difficult to break, and hence, *V*_*reset*_ increased with Ag dopant wt%.

**Figure 3:**
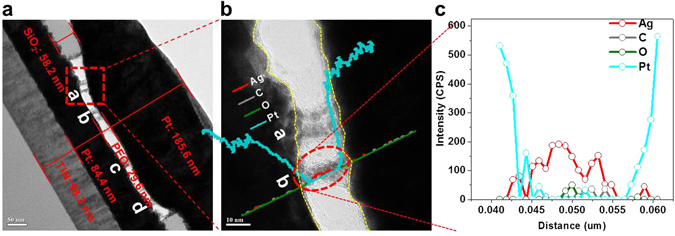
Morphology and chemical composition of Ag filaments in PEO polymer electrolyte. (**a**) cross-sectional TEM image of the CBRAM cell fabricated with 30 wt% Ag-doped PEO polymer electrolyte, (**b**) calibration between morphology and chemical compositional profile of Ag filament, and (**c**) chemical compositional depth profile of Ag filament. (*V*_*set*_), reset voltage (*V*_reset_), and memory margin (*I*_*on*_ */I*_*off*_).

The morphology of Ag filaments in PEO polymer electrolyte was investigated by x-TEM observation and chemical composition profile analysis using EDS. Note that the voltage scanning from 0 to V_set_ at the C.C of 10^−3^ A was applied on the CBRAM cell before the TEM observation. Figure 3 shows an x-TEM image of a PEO-based CBRAM cell doped with 30 wt% Ag after applying a set bias. The Ag filaments in the memory-cell region are cylindrical (a in Fig. 3b) or conical (b in Fig. 3b) and had a diameter of ~20.6 (a in Fig. 3a), ~18.4 (b in Fig. 3a), ~22.5 (c in Fig. 3a), and ~16.2 nm (d in Fig. 3a), indicating that several nanoscale filaments were produced during the set bias (voltage scanning from 0 to *V*_*set*_). The chemical composition of the filaments was analyzed by EDS and found to be Ag, C, and O, as shown in Fig. 3b,c. Since C and O originated from the electrolyte, it is evident that the filaments bridging the Pt cathode and anode in Fig. 3b are Ag filaments. This result implies that the randomly dispersed Ag ions in the PEO polymer electrolyte drift and diffuse from the top Pt cathode to the bottom Pt anode when a positive bias (*V*_*set*_) is applied and then produce Ag filaments in the electrolyte; this phenomenon will be examined in detail below.

**Figure 4:**
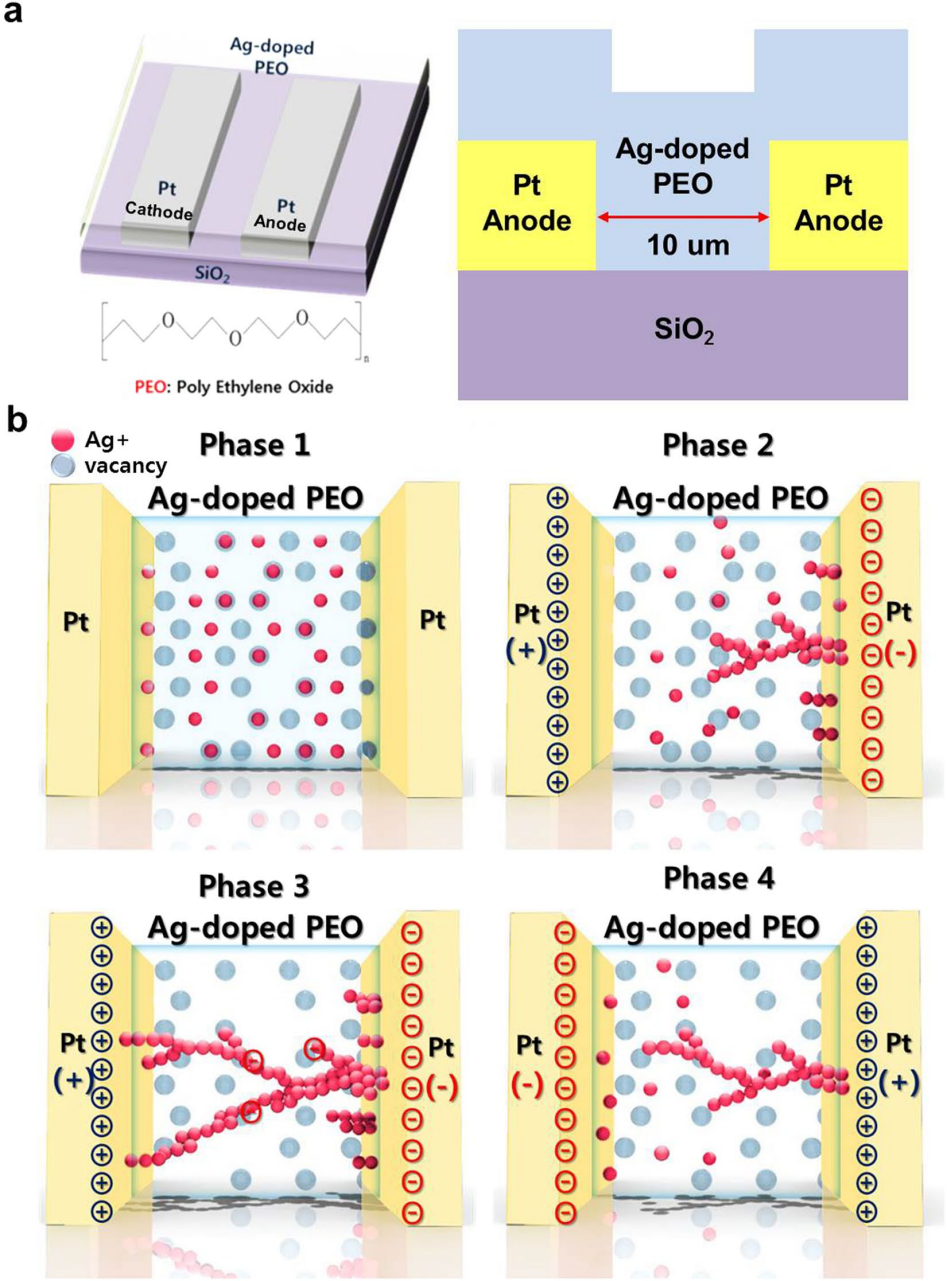
Lateral CBRAM cell with Ag-doped PEO polymer electrolyte between inert Pt electrodes. (**a**) Schematic device structure and (**b**) schematic drawing of electro-forming (set bias) and electro-breaking (reset bias) of Ag filaments in CBRAM cell.

**Figure 5:**
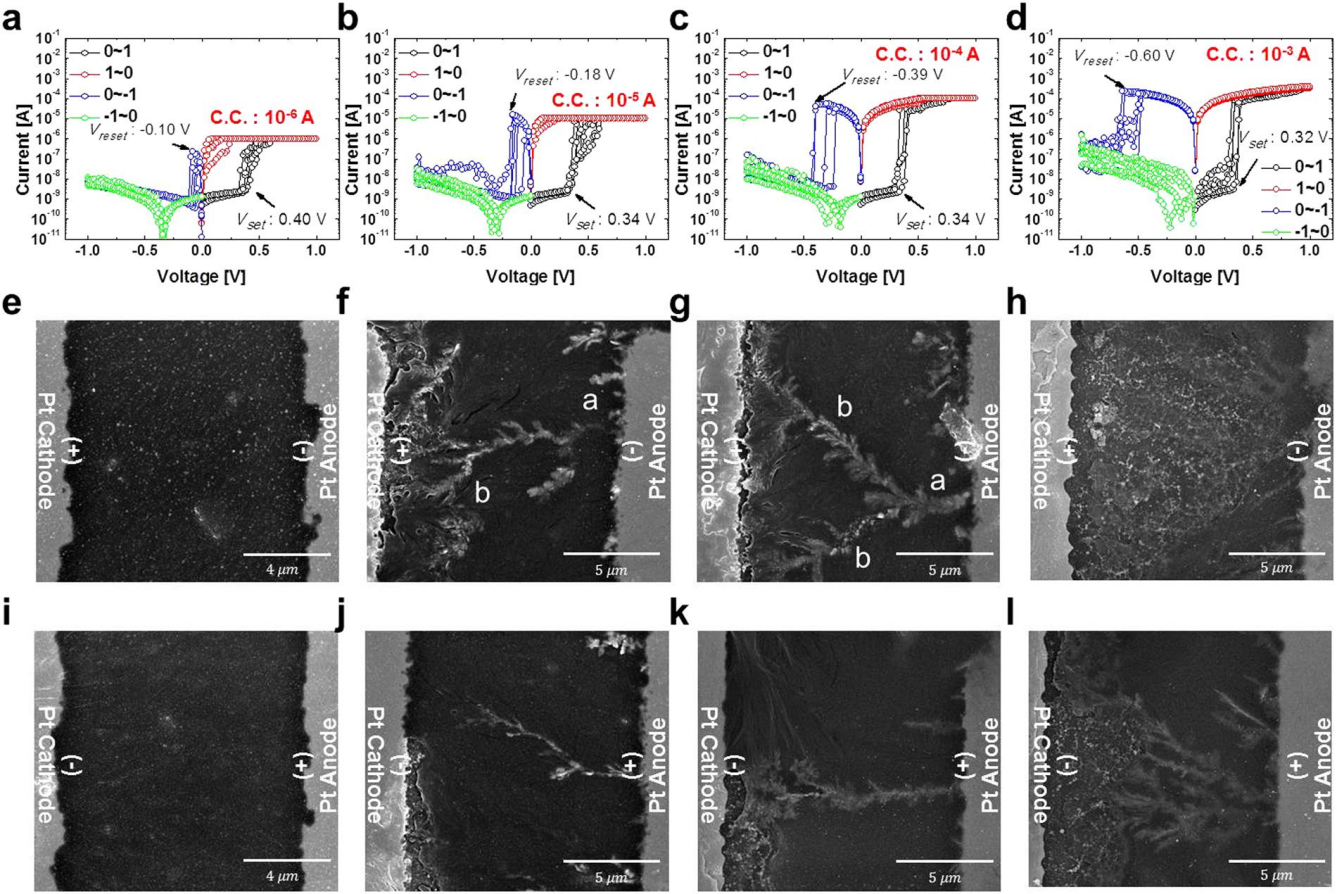
Dependence of bipolar switching characteristics and Ag filament morphology on compliance current level (C.C.). *I*–*V* curves for C.C. of (**a**) 1 × 10^−6^ A, (**b**) 1 × 10^−5^ A, (**c**) 1 × 10^−4^ A, and (**d**) 1 × 10^−3^ A. Ag filament morphologies after electro-forming process (set bias) for C.C. of (**e**) 1 × 10^−6^ A, (**f**) 1 × 10^−5^ A, (**g**) 1 × 10^−4^ A, and (**h**) 1 × 10^−3^ A. Ag filament morphologies after electro-breaking process (reset bias) for C.C. of (**i**) 1 × 10^−6^ A, (**j**) 1 × 10^−5^ A, (**k**) 1 × 10^−4^ A, and (**l**) 1 × 10^−3^ A.

Unlike previous CBRAM cells^7, 11–19^, the PEO-based CBRAM doped with Ag ions does not need an electro-forming process for bipolar switching because several Ag filaments are produced after application of the set bias, as shown in Fig. 3. However, the presence of Ag filaments in the electrolyte itself would be difficult to understand directly how the filaments are produced during the set bias or how the filaments are broken during the reset bias because of a nanoscale polymer electrolyte thickness (i.e., 29.6 nm). Thus, in order to understand the forming and breakage mechanisms of Ag filaments in the polymer electrolyte, we intentionally designed a CBRAM cell with a lateral device structure in which Ag-doped PEO polymer electrolyte was in the area between the Pt cathode and anode (the distance between the electrodes was 10 um (Fig. 4a)^28^. Note that 10 um would be enough long to observe the morphology of Ag filaments in the polymer electrolyte by using SEM. Then, we used it to investigate the dependence of the bi-polar switching on C.C. As shown in Fig. 5a–d, when C.C. increased from 1 × 10^−6^ to 1 × 10^−3^ A, *V*_*set*_ slightly decreased from +0.40 to +0.32, while *V*_*reset*_ increased from −0.10 V to −0.60 V and HRS increased from 1 × 10^−9^ to 4 × 10^−9^ A. To understand these results, we investigated the detailed morphology of the Ag filaments in the electrolyte in relation to C.C. (Fig. 5e–l).

Top-view SEM images and EDS analysis were used to examine the morphology of the Ag filaments after a set bias (i.e., voltage scanning from 0 to 1.0 V). At a C.C. of 1 × 10^−6^ A, no Ag filaments in the PEO polymer electrolyte were found and only Ag nanoparticles (i.e., white spots in the SEM image) were detected in the chemical composition analysis using EDS. This indicates that the dimensions of the Ag filaments producing current conduction paths after the set bias would be less than the dimension detection limit (Fig. 5a,e). When C.C. increased from 1 × 10^−6^ to 1 × 10^−5^ A, an Ag filament with primary a and secondary b dendrite structures was formed in the electrolyte and the filament bridged the cathode and anode so that the conduction current of the CBRAM cell rapidly increased from 2 × 10^−9^ A (HRS) to 1 × 10^−5^ A (C.C.), as shown in Figs 5b,f and S6a and e. As C.C. increased (from 1 × 10^−5^ to 1 × 10^−4^ A), more and more filaments grew and the diameters of the primary (a) and secondary (b) dendrites increased so that two secondary dendrites grew from the primary dendrite and eventually bridged at Pt cathode, thereby increasing the conduction current quickly from 3 × 10^−9^ A (HRS) to 1 × 10^−4^ A (C.C), as shown in Figs 5c,g and S6b and f. When C.C. increased even further (from 1 × 10^−4^ to 1 × 10^−3^ A), primary, secondary, and tertiary Ag dendrites grew and met one another such that the Ag filament looked like a conical bush^6^, and several dendrites bridged the cathode and anode; this resulted in an abrupt increase in current from 4 × 10^−9^ A (HRS) to 1 × 10^−3^ A (C.C), as shown in Fig. 5d,h. Figure 5e–h show that the number of Ag dendrites bridging at the Pt cathode increased with C.C., implying that *V*_*reset*_ slightly increases with C.C. In addition, the dependence of the Ag-filament morphology on C.C. indicates that the growth time of the filament would be less than 450 ms since the step time to increase 0.05 V was 56 ms. Note that the set time of a typical CBRAM cell using Ag or Cu ions is several dozen nanosecond^6, 29^.

Next, the morphology of the Ag filaments was investigated after applying the reset bias following the set bias, (i.e., voltage scanning from 0 to 1.0, 0, and-1.0 V). At a C.C. of 1 × 10^−6^ A, it was difficult to see the morphology of the filaments in the electrolyte; only Ag nanoparticles were found (Fig. 5a,e,i). As C.C. increased from 1 × 10^−6^ to 1 × 10^−5^ A, the primary dendrite of the Ag filament broke near the cathode through the oxidation of Ag ions in the dendrite near the Pt cathode and the drift of positively charged Ag (Ag^+^) ions toward the Pt cathode^4^, resulting in an increase in *V*_*reset*_ from −0.10 to −0.18 V(Figs 5b,f,j and S6c and g). As C.C. continued to increase (from 1 × 10^−5^ to 1 × 10^–4^ A), two secondary Ag dendrites that had grown from the primary dendrite broke near the Pt cathode as a result of the oxidation of Ag ions in the secondary dendrites and the drift of Ag^+^ ions toward the Pt cathode; this increased *V*_*reset*_ from −0.18 to −0.39 V (Figs 5c,g,k and S6d and h). Upon increasing C.C. even further (from 1 × 10^−4^ to 1 × 10^−3^ A), several dendrites of the tree-shaped Ag filament broke near the Pt cathode, thereby increasing *V*_*reset*_ from −0.39 to −0.60 V (Fig. 5d,h,l). Figure 5i–l obviously show that absolute value of *V*_*reset*_ increased with C.C. since the number of broken Ag dendrites of the filament near the cathode increased with C.C.

The morphology of the Ag filaments in the PEO polymer electrolyte in lateral-structure CBRAM cells in Fig. 5f let us understand that in 250-nm-diameter CBRAM cells in Fig. 3a. In lateral-structure CBRAM cells, many small Ag dendrites (arrows in Fig. 5f) were initially produced near the Pt anode when a positive bias was applied and then the primary Ag dendrite was continuously grown among many small Ag dendrites, followed by the growth of secondary and tertiary Ag dendrites, when the applied positive bias was increased up to *V*_*set*_, since the distance between the Pt anode and cathode (10 um) was very long, as shown in Fig. 5f. Otherwise, in 250-nm-diameter CBRAM cells, only many small Ag dendrites without the secondary and tertiary Ag-dendrite growth were produced between the Pt cathode and anode when the applied positive bias was increased up to *V*_*set*_, since the distance between the Pt cathode and anode (29.6 nm) was very short, as shown in Fig. 3a.

The dependence on C.C. of the morphology of the Ag filaments in the PEO polymer electrolyte can clearly explain the electro-forming and electro-breaking mechanism of the Ag filament, as shown in Fig. 4b. The blending of the AgNO_3_ solution with the PEO polymer solution produces singly positively charged Ag (Ag+) ions in the polymer via equation (1) below and as shown in Fig. 4b-phase 1;

1 $${{\rm{AgNO}}}_{3}\to {{\rm{Ag}}}^{+}+{{\rm{NO}}}_{3}^{-}$$

When a positive bias is applied to the Pt cathode and a negative bias is applied to the Pt anode, Ag+ ions drift and diffuse toward the anode, motivated by the applied electric field between the cathode and anode via an ion conduction process in the polymer electrolyte. When the Ag+ ions reach the anode, they are subject to a reduction process wherein the anode supplies electrons (equation (2)).

2 $$A{g}^{+}+{e}^{-}\to Ag$$

As a result, the primary Ag dendrites grow at a specific anode position where the highest electric filed is probably applied, as shown in Fig. 4b-phase 2. Note that the filaments grew at locally thin positions of the PEO polymer-electrolyte film, as shown Fig. 3a. When the applied bias increases further, the primary Ag dendrites create secondary and tertiary dendrite growth and eventually produce a filament bridging the cathode and anode, which acts a current conduction path, called electro-forming after a set bias, as shown in Figs 4b-phase 3 and 5g. In addition, as shown in Figs 4b-phase 4 and 5k, the Ag filaments are broken by the oxidation of Ag ions in the secondary dendrites and the drift of Ag^+^ ions toward the Pt cathode, when a negative bias is applied at the cathode, called electro-breaking after a reset bias. Therefore, Ag filaments bridging Pt cathode and anode grow from the Ag dendrite on the Pt anode via ionic conduction and reduction when a positive bias is applied to the Pt cathode, whereas Ag filaments near the cathode break through the oxidation of Ag ions in the secondary dendrites and the drift of Ag^+^ ions toward the Pt cathode, when a negative bias applied to the cathode.

**Figure 6:**
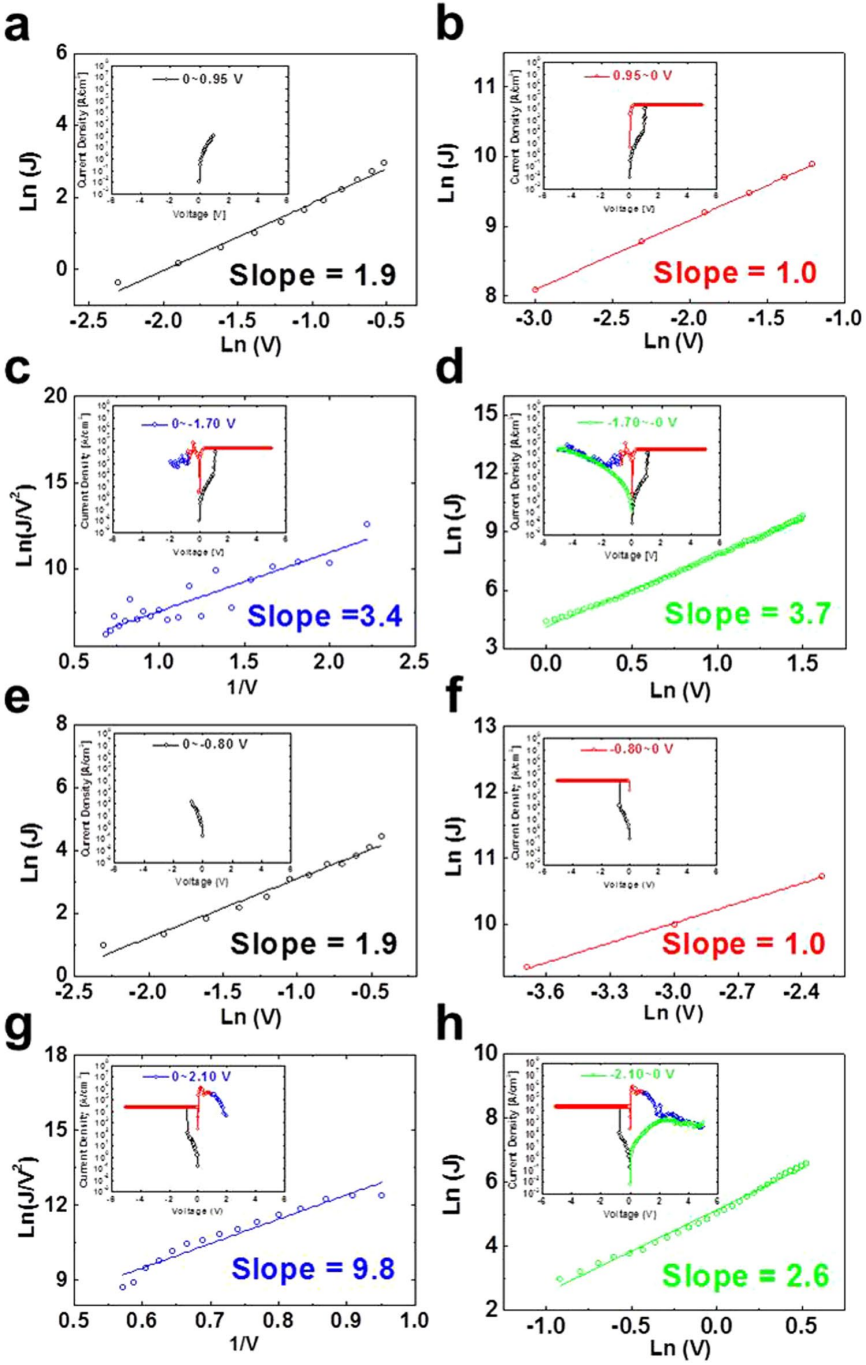
Current conduction mechanism for Ag-doped PEO polymer-electrolyte based CBRAM cell. For electro-forming at a positive applied bias on Pt cathode: (**a**) HRS between 0 and *V*_*set*_ (+0.95 V), (**b**) *V*_*set*_ (+0.95 V) and 0 V, (**c**) NDR, and (**d**) *V*_*reset*_ (−1.70 V) and 0 V. For electro-forming at a negative applied bias on Pt cathode: (**e**) HRS between 0 and *V*_*set*_ (−0.80 V), (**f**) *V*_*set*_ (−0.80 V) and 0 V, (**g**) NDR, and (**h**) *V*_*reset*_ (−2.10 V) and 0 V.

To understand the current conduction mechanism for the electro-forming and electro-breaking mechanism of the Ag filaments, the current conduction mechanism of the cell was examined by fitting the *I–V* curve when the applied bias was scanned at 0, 5, 0, −5, and 0 V. Four different *I–V* regions are present for electro-forming at a positive applied bias on the Pt cathode. The *I–V* curve of the HRS between 0 V and *V*_*set*_ (0.95 V) was well fitted by the ionic conduction mechanism with a slope of 1.9 (defined by equation (3) and shown in Fig. 6a ^6, 29^.

3 $$J\propto V/Texp(-d^{\prime} /T)$$

Here, *d’*, *V*, and *T* are a constant, the applied voltage, and the temperature, respectively. This fitting implies that Ag^+^ ions drift toward the Pt anode in the PEO polymer electrolyte by a positive applied electric field between the cathode and anode, i.e., via the current conduction mechanism of ion conduction, and eventually Ag filaments form between the cathode and anode by dendrite growth via reduction, called electro-forming after a set bias, as shown in Figs 4b-phase 2 and 3 and 6a. In addition, the *I*–*V* curve from 0.95 (*V*_*set*_) to 0 V fitted the Ohmic conduction mechanism (equation (4)).

4 $$J\propto Vexp(-c/T)$$

Here, *c*, *V*, and *T* are a constant, applied voltage, and temperature, respectively, indicating that current flows through Ag filaments in the electrolyte, as shown Figs 4b-phase 3 and 6b ^6, 30^. Furthermore, the *I*–*V* curve from 0 V to *V*_*reset*_ (−1.7 V) shows typical behavior of negative differential-resistance (NDR) that implies breakage of the filaments by the oxidation of Ag ion in the dendrite and drifting Ag^+^ ions toward the cathode due to the negative applied bias on the cathode, called electro-breaking after a reset bias, as shown in Figs 4b-phase 4 and 6c. Breakage of the dendrite occurred at near the Pt cathode since the diameter of the secondary and tertiary dendrites near the Pt cathode was smaller than that of the primary Ag dendrite grown on the Pt anode. The *I*–*V* curve of NDR was correlated with the current conduction mechanism of tunneling^6, 31^, as defined by equation (5) and shown in Fig. 6c.

5 $${\rm{J}}\propto {{\rm{V}}}^{2}\exp (-{\rm{b}}/{\rm{V}})$$

Here, *b* and *V* are a constant and the applied voltage, respectively, and as shown in Fig. 6c, the equation indicates that current flowing through Ag filaments in the electrolyte decreases rapidly. Finally, after the filaments break, the *I*–*V* curve from −1.70 to 0 V was fitted by the current conduction mechanism of ionic conduction with a slope of 3.7 (Fig. 6d). The slope of ionic conduction after the reset bias (bias scanning from −1.70 to 0 V), 3.7, was steeper than that after the set bias (voltage scanning from 0 to +0.95 V), 1.9, indicating that the absolute magnitude of *V*_*reset*_ (−1.70 V) was higher than that of *V*_*set*_ (0.95 V). Remind that the difference of the fitting slope of Fig. 6a,d would be originated from the difference between reduction and oxidation process of Ag ions. Therefore, the current conduction mechanism was described by the electro-forming and breaking process of the Ag filaments in the PEO polymer electrolyte between the cathode and anode.

For electro-forming at a negative applied bias on the Pt cathode, the *I*–*V* curves of four regions, i.e., 0 to *V*_*set*_ (−0.80 V), −0.80 to 0 V, NDR from 0 to *V*_*reset*_ (+2.10 V), and *V*_*reset*_ to 0 V, fitted ionic, Ohmic, tunneling, and ionic conduction, respectively, which is the same as the current conduction mechanism for electro-forming at a positive applied bias on the Pt cathode and electro-breaking at a negative applied bias on the Pt electrode, as shown in Fig. 6e–h. These results mean that the same current conduction mechanism makes the Ag-doped PEO-based CBRAM cell capable of bipolar switching, regardless of the applied bias direction of electro-forming.

## Discussion

The CBRAM cells demonstrated an electro-forming-free behavior and their bipolar switching characteristics strongly depended on the Ag dopant concentration in the PEO polymer electrolyte. A higher Ag dopant concentration led to a lower *V*_*set*_, a higher *V*_*reset*_, and a higher memory margin (*I*_*on*_ */I*_*off*_). In particular, they were independent of the applied bias direction of electro-forming, which is completely different from conventional CBRAM cells. In addition, the morphologies of the Ag filaments, i.e., conical or cylindrical with diameters of 16.2~22.5 nm, in the PEO polymer electrolyte showed current conduction paths via electro-forming after a set bias was applied. Moreover, the dependency of the morphologies of the Ag filaments in the electrolyte in relation to the compliance current level clearly explained the mechanism of electro-forming after a set bias and electro-breaking after a reset bias. Electro-forming involved Ag^+^ ions drifting and diffusing toward the Pt anode in the polymer electrolyte under a positive applied bias on the Pt cathode and eventual Ag filament formation by reduction and dendrite growth. On the other hand, electro-breaking occurred through the oxidation of Ag ions in the secondary dendrites and the drift of Ag^+^ ions toward the Pt cathode. These results indicate that nanoscale Ag filaments only grow through primary Ag dendrites on the Pt anode and eventually reach the Pt cathode so that nanoscale conical or cylindrical Ag filaments are produced if the thickness of the PEO polymer electrolyte is nanoscale (i.e., 29.6 nm). The current conduction mechanism of the CBRAM cell was in accordance with electro-forming (after set bias) and electro-breaking (after reset bias) mechanism, i.e., electro-forming by ionic conduction and electro-breaking by tunneling conduction. Our results indicate that an electro-forming free CBRAM cell could be achieved with a sandwich structure consisting of an inert electrode (Pt, TiN, W, etc.), solid electrolyte (binary transition oxide: CuO, TiO_2_, Ta_2_O_5_, Al_2_O_3_, and HfO_2_ etc.), and inert electrode. In addition, the analysis of electro-forming and electro-breaking in the CBRAM cell suggests that the shape, diameter, and number of Ag filaments in the electrolyte can be controlled by varying the doping concentration of metallic ions in the electrolyte, which in turn determines the bi-polar switching characteristics of the cells.

## Methods

20 × 20 memory-cell arrays were fabricated with a nanoscale hole structure, 250 nm in diameter and 58 nm height, having a Pt cathode and anode, where the PEO polymer electrolyte thickness was ~29.6 nm and each memory cell was isolated by a SiO_2_ layer (Figs 1a and 3a). To investigate the dependence of the Ag dopant concentration on the bi-polar switching characteristics, AgNO_3_ was blended with PEO using a co-solvent of acetonitrile and ethanol (1:1 ratio) at 30 °C for 3 hrs. PEO is a polymer electrolyte for flexible CBRAM cells^32^. The blended Ag weight (wt) % concentration in the PEO layer was measured by UV-vis spectroscopy. The UV absorption peak of AgNO_3_ in PEO was detected at ~230 nm in wavelength, as shown in Fig. 1b. The absorbance of AgNO_3_ in PEO linearly increased with the AgNO_3_ wt% concentration, as shown in the inset of Fig. 1b, indicating that the UV absorbance of the Ag blended PEO film could accurately determines the AgNO_3_ wt% concentration. Then, Ag-doped PEO solutions at 0, 15, 30, 60 and 75 wt% were spin-coated on the memory-cell arrays at 2000 rpm for 120 s, and the arrays were then baked at 60 °C for 5 min to remove residual solvent. Afterward, the Pt cathodes, 300 μm in diameter and 200 nm in thickness, were deposited at a deposition rate of 2.44 Å/s at 10^−5^ Pa by using a shadow mask. Thus, the fabricated CBRAM cells had a vertical device structure consisting of a Pt cathode, Ag-doped PEO polymer electrolyte, and Pt anode.

To clarify the electro-forming and electro-breaking mechanism of the Ag filaments, cells with a lateral structure were intentionally fabricated, as shown in Fig. 4a. In this case, a 100-nm-thick Pt cathode and anode were patterned on 200-nm-thick SiO_2_ film that was thermally grown on a Si wafer, and they were laterally isolated by placing them 10 um apart. Then, Ag-doped PEO solutions at 15, 30, 60 and 75 wt% were spin-coated on the wafer. The resulting Ag-doped PEO CBRAM cells had a lateral device structure consisting of Ag-doped PEO polymer electrolyte between a Pt cathode and anode. The morphology and chemical composition of Ag filaments in the electrolyte were investigated by inspecting top-view SEM images and conducting an EDS analysis. The material properties of the PEO solid electrolytes were observed by UV-Vis-NIR Spectrophotometer (Carry 5000), TEM (JEM-2100F) and SEM (nova nano SEM 450). The electrical characteristics on temperature were measured using an Agilent B2902A semiconductor parameter analyzer.

## Electronic supplementary material

Supplementary Information
