# Supplementary material for: Electro-Forming and Electro-Breaking of Nanoscale Ag Filaments for Conductive-Bridging Random-Access Memory Cell using Ag-Doped Polymer-Electrolyte between Pt Electrodes
Source: Sci Rep. 2017 Jun 8;7:3065. doi: 10.1038/s41598-017-02330-x (PMC5465185; doi:10.1038/s41598-017-02330-x)

Supplementary Information for

**Electro-Forming and Electro-Breaking of Nanoscale Ag Filaments for Conductive-Bridging Random-Access Memory Cell using Ag-Doped Polymer-Electrolyte between Pt Electrodes**

**Myung-Jin Song, Ki-Hyun Kwon, and Jea-Gun Park ***

Department of Electronics and Computer Engineering, Hanyang University, Seoul, 04763, Republic of Korea

*parkjgl@hanyang.ac.kr

**Supplement 1. Dependence of bipolar switching characteristics on compliance current level(C.C.) for Ag-doped PEO polymer-electrolyte based CBRAM cells. *I-V curves* for** (**a**) 10^-6^ A, (**b**) 10^-5^ A and (**c**) 10^-4^ A at a positive applied bias and (**d**) 10^-6^ A, (**e**) 10^-5^ A and (**f**) 10^-4^ A at a negative applied bias.

***Supplement 1***

**
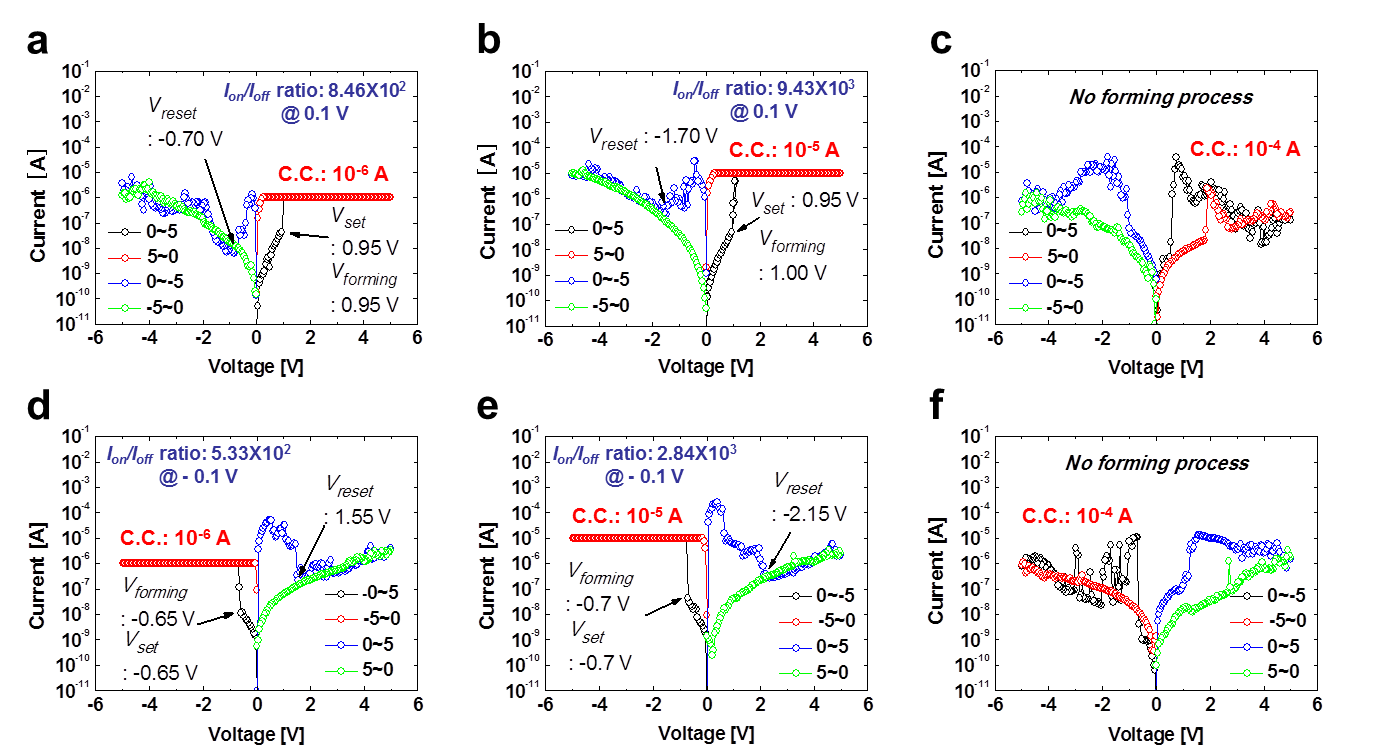
**

**Supplement 2. DC endurance cycles for 30 % Ag-doped PEO polymer-electrolyte based CBRAM cells at a positive applied bias.**

***Supplement 2***

**
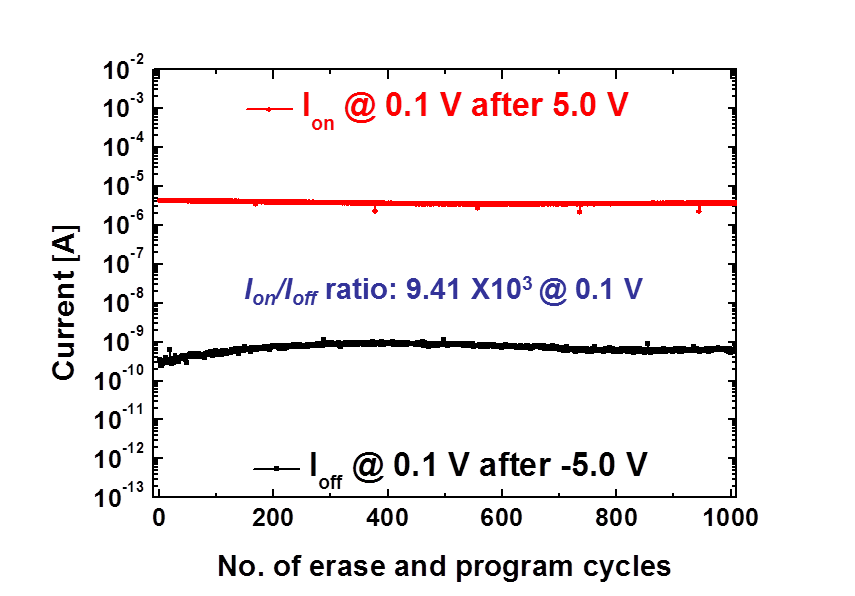
**

**Supplement 3. Cumulative distribution of switching characteristics. Under electroforming at a negative applied bias,** (**a**) *I_HRS_* and *I_LRS_* and (**b**) *V_forming_*, *V_set_*, and *V_reset_*. Under electroforming at a positive applied bias, (**c**) *I_HRS_* and *I_LRS_* and (**d**) *V_forming_*, *V_set_*, and *V_reset_*. Total number of the measured cells was 20.

***Supplement 3***

**
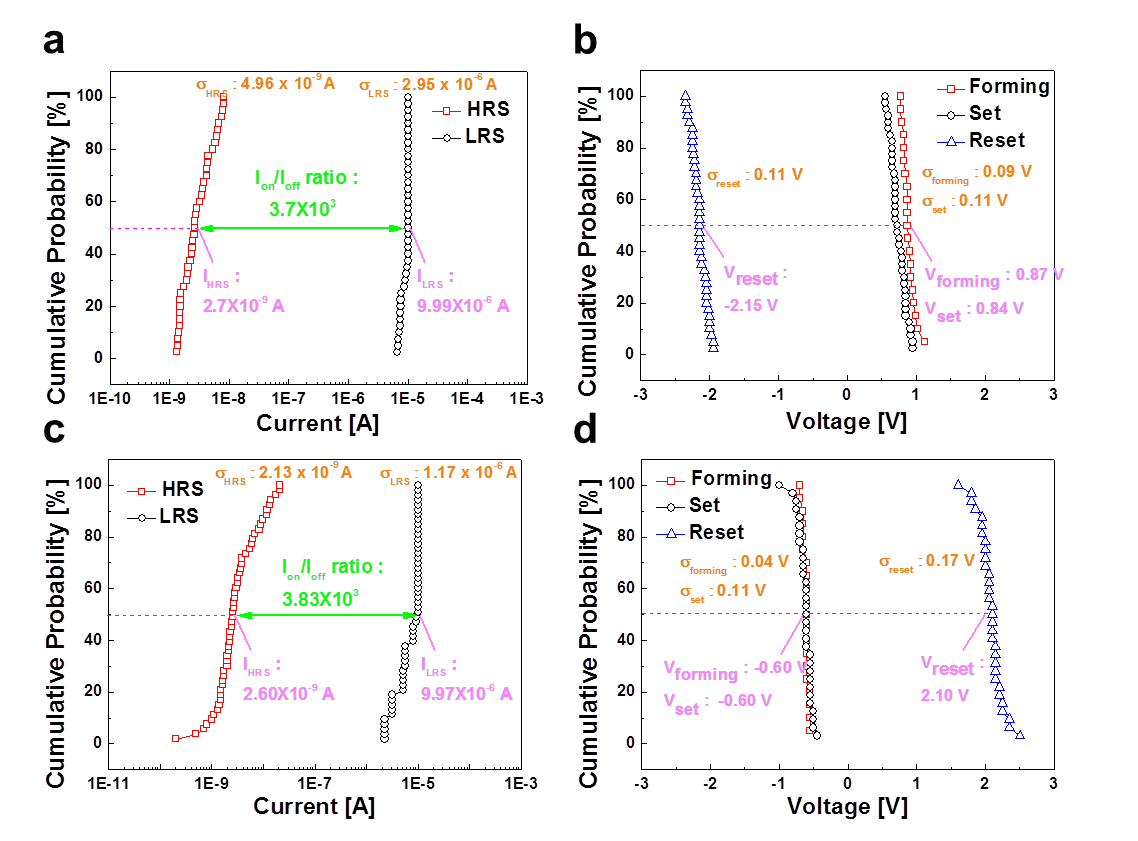
**

**Supplement 4. Dependence of bipolar switching characteristics on Ag doping concentration and the polarity of the applied bias for Ag-doped PEO polymer-electrolyte based CBRAM cells. Compliance current level (C.C.), set voltage (*V_set_*), reset voltage (*V*_reset_), and memory margin (*I_on_/I_off_* )** (**a**) at a positive applied bias and (**b**) at a negative applied bias. The bipolar switching characteristics at a negative applied bias was almost similar to that a at positive applied bias.

***Supplement 4***

**
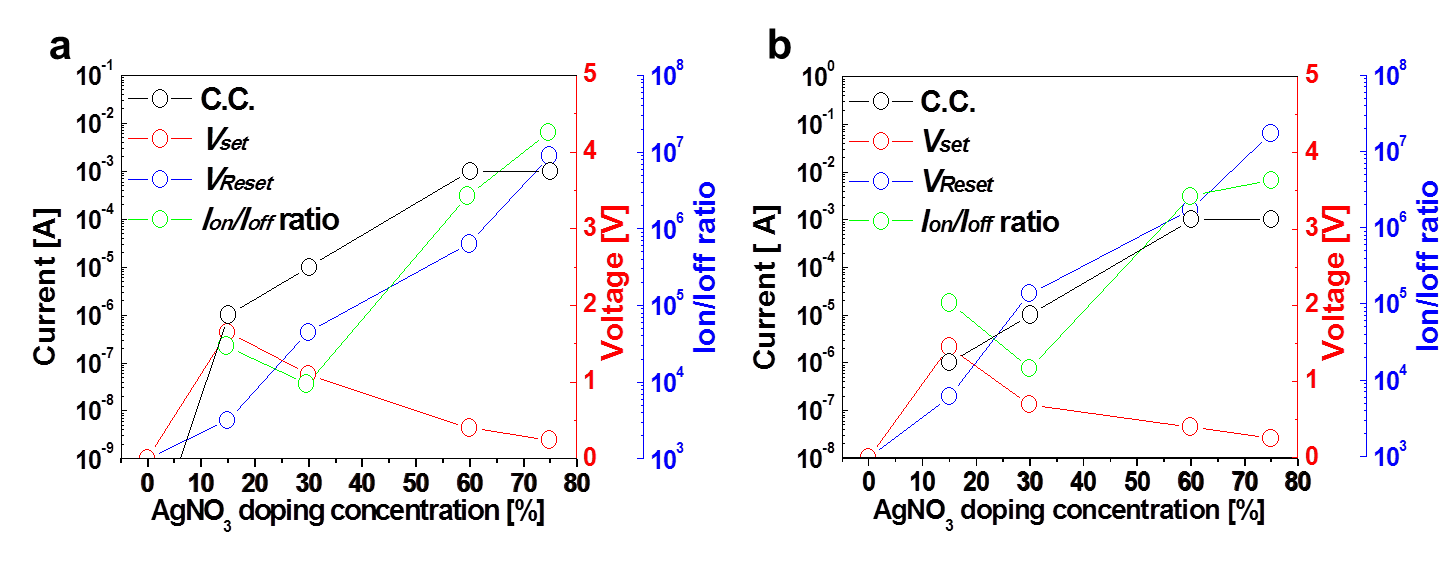
**

**Supplement 5. DC endurance cycles on compliance current level(C.C.) for 30 % Ag-doped PEO polymer-electrolyte based CBRAM cells at a negative applied bias.** (**a**) 10^-6^ A and (**b**) 10^-5^ A.

***Supplement 5***

**
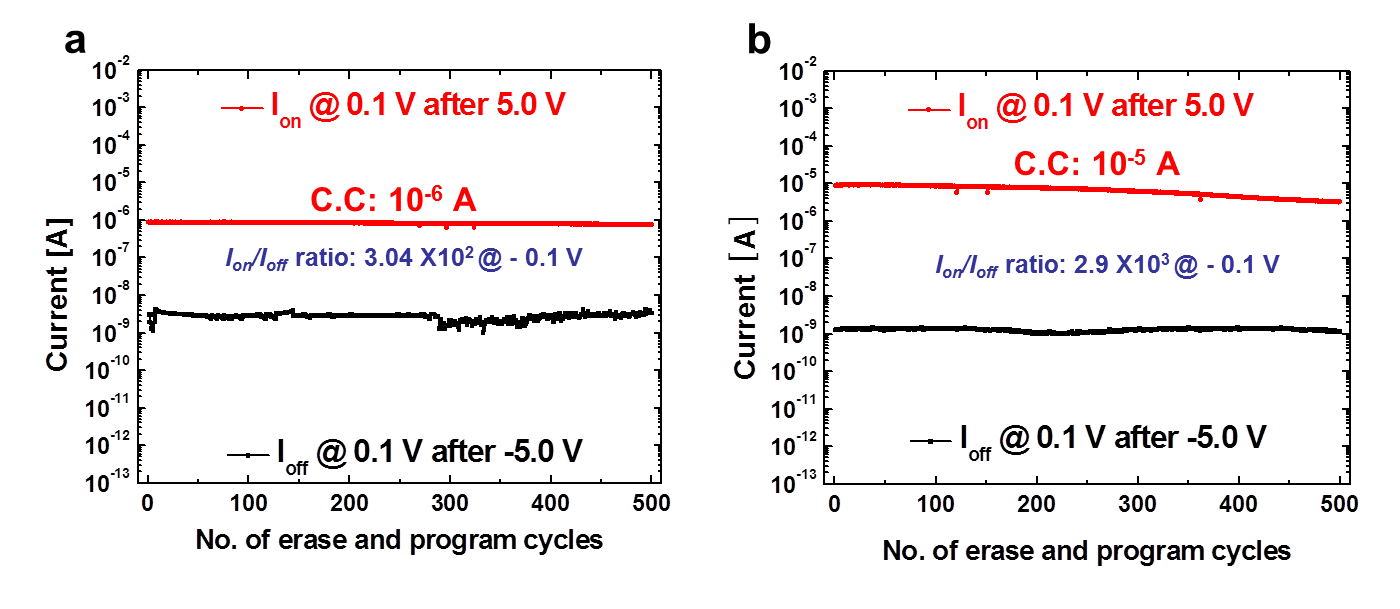
**

**Supplement 6. Correlation between morphology and chemical composition of Ag filaments for Ag-doped PEO polymer-electrolyte based CBRAM cells. Top-view SEM images after electro-forming process (set bias) for C.C. of** (**a**) 1x10^-5^ A and (**b**) 1x10^-4^A. Top-view SEM images after electro-breaking process (reset bias) for C.C. of (**c**) 1x10^-5^ A and (**d**) 1x10^-4^ A. EDX chemical composition analysis done after electro-forming process (set bias) for C.C. of (**e**) 1x10^-5^ A and (**f**) 1x10^-4^ A, where yellow spots correspond to Ag ions. EDX chemical composition analysis after electro-breaking process (reset bias) for C.C. of (**g**) 1x10^-5^ A and (**h**) 1x10^-4^ A.

***Supplement 6***


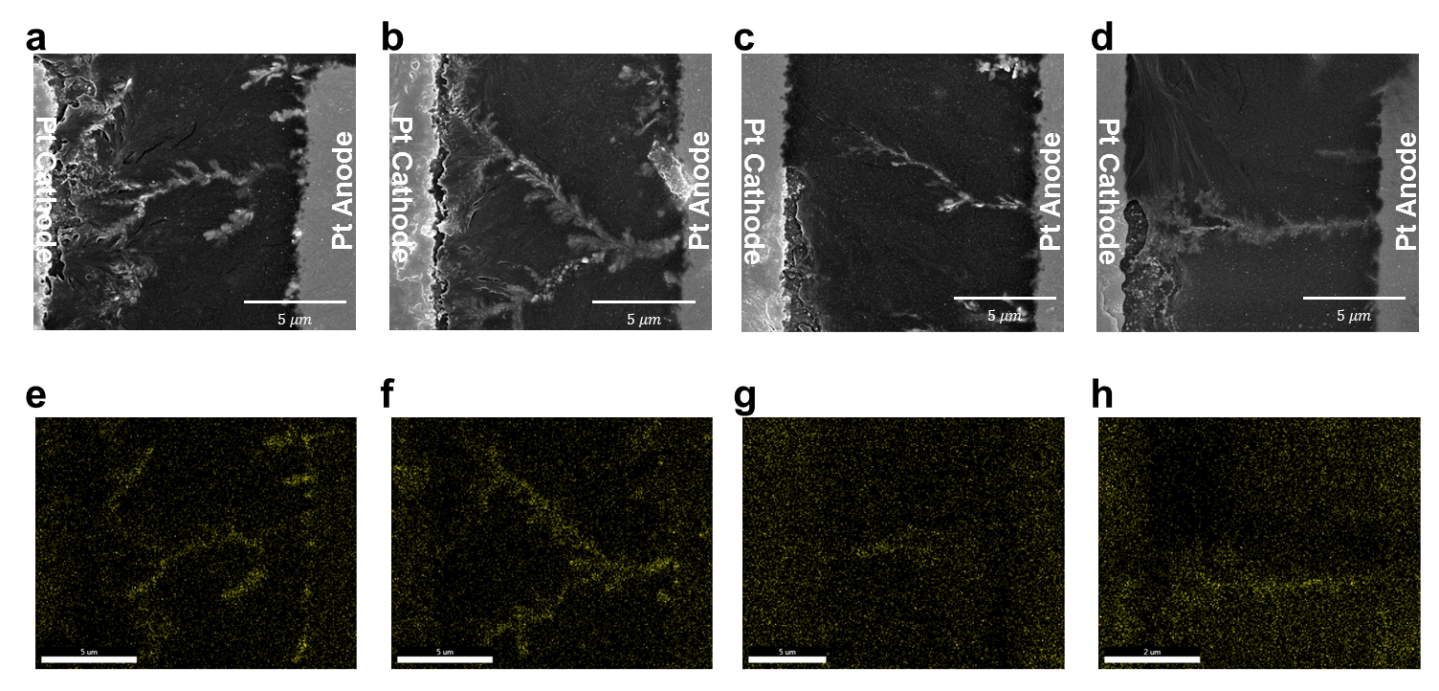

Supplement: Supplementary file 1 — Supplementary Information [file 41598_2017_2330_MOESM1_ESM.doc]
